# Excess visceral fat area as an independent risk factor for early postoperative complications in patients with obesity undergoing bariatric surgery

**DOI:** 10.3389/fendo.2023.1072540

**Published:** 2023-02-09

**Authors:** Liping Han, Chaoyi Deng, Rui Zhao, Qianyi Wan, Xiaofang Zhang, Xiao Wang, Yi Chen

**Affiliations:** ^1^ Department of Anesthesiology, West China Hospital, Sichuan University, Chengdu, China; ^2^ Laboratory of Anesthesia and Critical Care Medicine, National-Local Joint Engineering Research Center of Translational Medicine of Anesthesiology, West China Hospital, Sichuan University, Chengdu, China; ^3^ Department of Gastrointestinal Surgery, West China Hospital, Sichuan University, Chengdu, China

**Keywords:** obesity, bariatric surgery, laparoscopic sleeve gastrectomy, visceral fat area, postoperative complications

## Abstract

**Background:**

Few studies have investigated the correlation between visceral fat area (VFA) and early postoperative complications in patients with obesity undergoing bariatric surgery. This study aimed to investigate the relationship between VFA and early postoperative complications in patients with obesity following bariatric surgery.

**Methods:**

The study was conducted at a tertiary university hospital. Patients with obesity who underwent laparoscopic sleeve gastrectomy between June 2016 and October 2020 were divided into two groups based on umbilical level VFA: high-VFA group (umbilical level VFA ≥ 100 cm^2^) and low-VFA group (umbilical level VFA < 100 cm^2^). Baseline characteristics, intraoperative and postoperative conditions, and early postoperative complications were compared between the groups. The primary outcome was early postoperative complications, and the secondary outcome was postoperative hospital stay.

**Results:**

The study included 152 patients, with 82 patients in the low-VFA group and 70 patients in the high-VFA group. The high-VFA group had a higher incidence of early postoperative complications (14.29% vs. 2.44%, P = 0.013) than the low-VFA group. The length of postoperative hospital stay did not differ significantly between the groups.

**Conclusions:**

Our study suggests that excess VFA is an independent risk factor for early postoperative complications following bariatric surgery, and VFA may be used in preoperative evaluations.

## 1 Introduction

Obesity is a chronic disease that is traditionally defined as an excess of body fat that causes health prejudice. The prevalence of obesity has rapidly increased and is now considered as a global epidemic (1). Obesity increases the risk of developing a variety of metabolic disorders, such as type 2 diabetes, hyperlipidemia, obstructive sleep apnea (OSA), hypertension and other cardiovascular diseases, and cancer, resulting in a decrease in life expectancy and quality of life (2–5). The risk of complications associated with obesity is proportional to the degree of obesity and, more importantly, fat accumulation (6). Bariatric surgery is considered the most effective long-term treatment for severe obesity. For patients with obesity, bariatric surgery is associated with a longer life expectancy than conventional bariatric care (7). Bariatric surgery can not only achieve satisfactory weight loss but also reduce the risk of cardiovascular disease, type 2 diabetes, and cancer-related diseases (8). The development of minimally invasive surgery and laparoscopic techniques has made laparoscopic sleeve gastrectomy (LSG) the mainstream surgical method for metabolic surgery (9). LSG has fewer complications than some other bariatric procedures such as laparoscopic Roux-en-Y gastric bypass owing to the simple and feasible procedure, maximizing the preservation of changes to normal anatomy (10).

The body mass index (BMI) is currently the most widely used metric for assessing obesity. Although a correlation exists between BMI and human metabolic health, it is not sensitive to body fat distribution and does not accurately reflect the severity of obesity (11). The waist-to-hip ratio is a simple estimate of visceral obesity but cannot distinguish between subcutaneous and visceral adipose tissue (12). Magnetic resonance imaging and computed tomography (CT) quantitatively measure the patterns of body fat distribution and are reliable criteria for diagnosing visceral adipose tissue (VAT) (13). Visceral fatty obesity is primarily characterized by excessive fatty tissue that fills and lines the abdominal cavity, organs, blood vessels, and other tissues. Visceral obesity is characterized by an umbilical level visceral fat area (VFA) ≥ 100 cm^2^ (14).

VFA is associated with a poor prognosis in gastrointestinal surgery (15, 16). However, there are few studies on the impact of VFA on bariatric surgery and perioperative management in patients with obesity. This retrospective study was aimed to investigate the relationship between VFA and early postoperative complications in patients with obesity following bariatric surgery.

## 2 Materials and methods

### 2.1 Study design and patients

This single-center, retrospective study used data from the discharge medical records of patients undergoing gastrointestinal surgery in our institution’s Department of Gastrointestinal Surgery from June 2016 to October 2020. All research procedures involving human participants meet the ethical standards of the institutional research council. This study was approved by our institution’s ethics committee and registered at www.chictr.org.cn (ChiCTR2200062041).

### 2.2 Inclusion and exclusion criteria

The inclusion criteria were as follows: aged 16–65 years; BMI ≥ 28 kg/m^2^; undergoing selective LSG under general anesthesia. Patients were excluded if they did not have multi-row spiral abdominal CT fat volume scans; if they had other chronic diseases such as tumors, chronic kidney disease, or inflammatory bowel disease; if they were addicted to drugs or alcohol or had an uncontrollable mental illness; if they were taking glucocorticoids or sex hormones; or if they had American Society of Anesthesiologists (ASA) status V–VI.

### 2.3 Data collection

The collected data included the following: patient characteristics (age, sex, height, total body weight [= actual weight], BMI, ASA status, lean body weight [calculated based on height and weight], and visceral–subcutaneous ratio [VSR]); underlying diseases, including hypertension, hepatic steatosis, OSA syndrome, and diabetes; surgical data (surgery time, postoperative hospital stay, and postoperative adverse reactions); abdominal CT scan data; and anesthesia-specific data (anesthesia method, duration of anesthesia, anesthesia monitoring, anesthesia drugs [opioids, hypnotics, vasoactive drugs, and muscle relaxants and their antagonists], volume displacement [crystalline, colloidal], urine output, and anesthesia records).

### 2.4 Perioperative strategies

All enrolled patients received perioperative care in bariatric surgery according to the Enhanced Recovery After Surgery (ERAS) guidelines (17). Patients fasted for 8 h before the operation, and the intestinal tract was well prepared. General anesthesia was selected as the anesthesia method. All patients were treated using a high-definition laparoscopic surgery system.

### 2.5 Evaluation of visceral obesity

**Figure 1:**
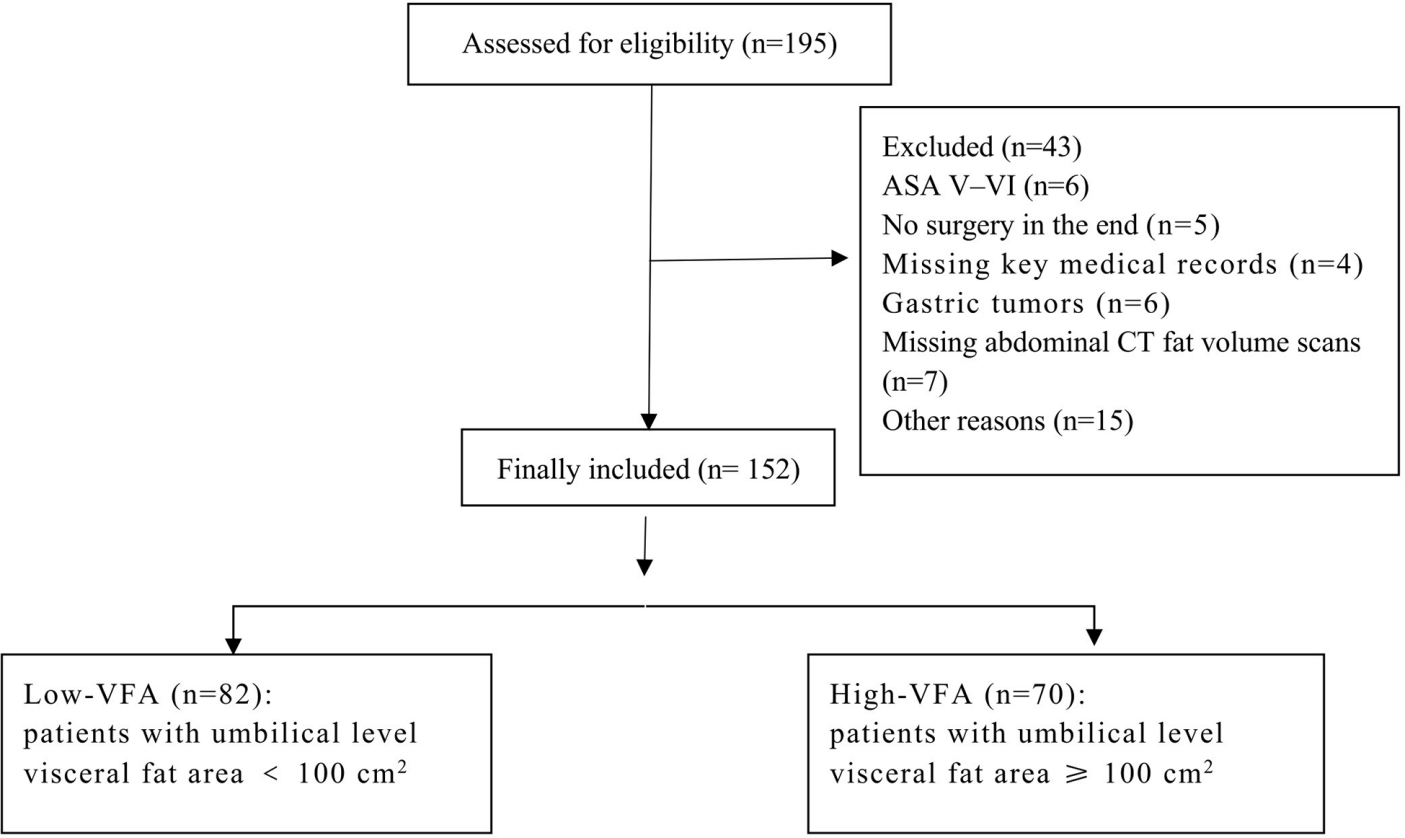
Flowchart of the patient selection process. ASA, American Society of Anesthesiologists.

Patients undergoing multi-row spiral CT (Siemens products) were instructed to hold their breath during the scan. The scanning range was from the diaphragm to the pelvic floor, which was reconstructed using the B30f algorithm. We measured the umbilical fat and skeletal muscle areas on the available CT scan images. The edges of skeletal muscle and adipose tissue at this layer were manually delineated on a GE ADW 4.6 workstation using the syngo MultiModality Workplace software (Siemens Healthineers AG, Forchheim, Germany). The CT values for skeletal muscle and adipose tissue were set at -29 to 150 HU and -150 to -50 HU, respectively; the software calculated the area of each part within the set range (18). According to the Japan Society for the Study of Obesity and widely accepted clinical criteria, the threshold for visceral adiposity measured at the umbilical level on CT images is 100 cm^2^. Patients with umbilical level VFA ≥ 100 cm^2^ were considered to have high-VFA, whereas those with VFA < 100 cm^2^ were considered to have low-VFA (**Figure 1**).

### 2.6 Definition of complications

Early postoperative complications were defined as complications that occurred within 30 days following surgery, including anastomotic leakage, intestinal obstruction, infectious complications (e.g. pneumonia and wound infection), and cardiovascular events (19). The severity was graded using the Clavien–Dindo classification (20). The primary outcome of bariatric surgery was early postoperative complications, and the secondary outcome was postoperative hospital stay. Physicians were unaware of which group the patients had been divided into when diagnosing complications.

### 2.7 Statistical analysis

Statistical calculations were performed using SPSS software version 26.0 (SPSS Inc., Chicago, IL). Continuous variables are expressed as mean ± standard deviation; categorical variables are expressed as numbers or percentages (%). Intergroup comparisons of numerical variables were performed using one-way analysis of variance and logistic regression analysis, chi-square tests, or Fisher precision tests for the comparison of classified data. Logistic regression analysis was used to analyze the relationship between postoperative complications and various variables. Potential confounding variables (p < 0.05) and known clinically significant variables such as age (p < 0.1) were included in the multivariate analysis. The forward stepwise regression approach was used in the multiple logistic regression model. p < 0.05 indicates statistical significance.

## 3 Results

### 3.1 Comparison of baseline characteristics

In total, 152 patients with obesity who underwent LSG at our institution were enrolled. The patients were 62.5% women, with a mean age of 33.59 ± 9.72 years (range 16–64 years) and a mean BMI of 36.97 ± 5.12 kg/m^2^ (range 28.01–57.14 kg/m^2^). The patients were divided into two groups based on a VFA of 100 cm^2^: high- (n = 70) and low- (n = 82) VFA groups. We compared the baseline characteristics of the groups; the results are presented in **Table 1**. The groups were comparable in terms of ASA scores, type 2 diabetes, and hepatic steatosis. The groups significantly differed in terms of sex (p = 0.001), mean age (p = 0.007), height (p = 0.028), weight (p < 0.001), BMI (p = 0.014), lean body mass (p < 0.001), total fat area (p < 0.001), abdominal wall fat area (p < 0.001), VSR (p < 0.001), skeletal muscle area (p < 0.001), hypertension (p = 0.021), hyperlipidemia (p = 0.011), and sleep apnea-hypopnea syndrome (p = 0.045). Compared to the low-VFA group, the high-VFA group had a higher incidence of postoperative complications (p = 0.013; **Table 2**).

**Table 1:** Baseline characteristics of low-VFA vs. high-VFA groups.

	Low-VFA (n=82)	High-VFA (n=70)	*p*-value
Sex (male/female)	20/62	37/33	0.001^*^
Age (yrs)	31.29 ± 0.97	36.29 ± 1.21	0.007^*^
ASA (I/II/III/IV)	3/64/15/0	1/47/20/2	0.14^*^
Anthropometric parameter			
Height (cm)	163.98 ± 0.89	167.31 ± 1.00	0.03^*^
Total body weight (kg)	97.03±1.85	107.24 ± 2.34	< 0.001^*^
BMI (kg/m^2^)	35.92 ± 0.47	38.20 ± 0.69	0.01^*^
Lean body mass (kg)	54.01 ± 1.19	61.07 ± 1.34	< 0.001^*^
Total fat area (cm^2^)	212.88 ± 7.39	338.26 ± 12.29	< 0.001^*^
Abdominal wall fat area (cm^2^)	138.98 ± 6.15	197.81 ± 9.75	< 0.001^*^
VSR	0.63 ± 0.04	0.84 ± 0.06	< 0.001^*^
Skeletal muscle area (cm^2^)	65.84 ± 2.10	89.69 ± 2.56	< 0.001^*^
Comorbidities			
Hypertension (%)	11 (13.41)	20 (28.57)	0.02^*^
Type 2 Diabetes (%)	18 (21.95)	22 (31.43)	0.19
Hepatic steatosis (%)	43 (52.44)	33 (47.14)	0.52
Hyperlipidemia (%)	0 (0)	7 (10.00)	0.01^*^
OSA (%)	0 (0)	5 (7.14)	0.05

ASA, American Society of Anesthesiologists; BMI, Body Mass Index; OSA, obstructive sleep apnea syndrome; VFA, visceral fat area; VSR, visceral to subcutaneous fat ratio.

*statistically significant (P < 0.05).

**Table 2:** Perioperative management and outcomes of low-VFA vs. high-VFA groups.

	Low-VFA (n=82)	High-VFA (n=70)	*p*-value
Operation time (min)	104.23 ± 3.43	109.54 ± 5.07	0.33
Anesthesia time (min)	164.26 ± 4.50	164.35 ± 5.71	0.85
Fluid infusion (mL)	1071.43 ± 45.77	1240.74 ± 67.51	0.005^*^
Urine volume (mL/kg/h)	1.62 ± 0.24	1.32 ± 0.13	0.56
Sufentanil dosage (µg)	32.21 ± 0.89	35.00 ± 1.27	0.02^*^
Remifentanil dosage (µg)	1309.15 ± 60.70	1235.34 ± 62.73	0.99
Oxygen saturation under air inhalation (%)	98.60 ± 0.23	97.06 ± 0.37	< 0.001^*^
Intraoperative oxygen saturation below 95% of the time (min)	2.21 ± 0.65	7.06 ± 1.90	0.05
Postoperative hospital stay (days) M (P25, P75)	4 (3, 4)	4 (3, 5.25)	0.87
Postoperative complications (%)			0.013^*^
Grade I	0 (0)	3 (4.29)	
Grade II	1 (1.22)	3 (4.29)	
Grade III	1 (1.22)	3 (4.29)	
Grade IV	0 (0)	1 (1.43)	
Grade V	0 (0)	0 (0)	
≥ Grade III	1 (1.22)	4 (5.71)	0.86
Pulmonary infection (%)	0 (0)	1 (1.43)	
Abdominal infection (%)	0 (0)	3 (4.29)	
Gastrointestinal bleeding (%)	0 (0)	1 (1.43)	
Respiratory failure (%)	0 (0)	2 (2.86)	
Anastomotic leakage (%)	2 (2.44)	0 (0)	
Intestinal obstruction (%)	0 (0)	1 (1.43)	
Cardiovascular disease (%)	0 (0)	2 (2.86)	

M, median; P25, 25% quantile; P75, 75% quantile; VFA: visceral fat area.

*statistically significant (P < 0.05).

### 3.2 Risk factors for postoperative complications

A univariate analysis was conducted to identify risk factors for postoperative complications (**Table 3**). Gender, age, VFA, hypertension, OSA syndrome, skeletal muscle area, and infusion volume were included in the logistic multivariate regression analysis. Only VFA was statistically significant (p < 0.05), presenting as an independent risk factor for early postoperative complications.

**Table 3:** Univariate and multivariate analysis of factors associated with postoperative complications.

	Postoperative complications			
	Univariate analysis		Multi-factor analysis	
	OR (95%CI)	*p*-value	OR (95%CI)	*p*-value
Sex	0.17 (0.05–0.67)	0.01^*^		
Age (yrs)	1.04 (0.98–1.10)	0.08		
VFA (cm^2^)	1.02 (1.01–1.04)	< 0.001^*^	1.02 (1.01–1.04)	< 0.001^*^
Skeletal muscle area (cm^2^)	1.04 (1.01–1.06)	0.003^*^		
Hypertension (%)	3.13 (0.92–10.56)	0.07		
OSA	9.13 (1.37–61.12)	0.02^*^		
Intraoperative oxygen saturation < 95%	1.03 (1.01–1.06)	0.01^*^		

OSA, obstructive sleep apnea syndrome; VFA, visceral fat area.

*statistically significant (P < 0.05).

## 4 Discussion

In this single-center, retrospective cohort study, we used abdominal CT as an entry point, which is more accurate than BMI, to reflect the body fat distribution, and investigated the impact of visceral fat on early postoperative complications in patients who had undergone bariatric surgery. VFA may be a risk factor for early postoperative complications in patients with obesity undergoing bariatric surgery. The findings of this study add to the literature on the impact of visceral fat on prognosis in bariatric surgery and suggest that anesthesiologists focus not only on BMI but also on the patient’s body composition in anesthesia management of patients with obesity to achieve precise anesthesia. Because of the wide variations in the distribution of human adipose tissue, BMI cannot accurately reflect the degree of obesity in the visceral cavity. Our binary logistic multivariate regression analysis revealed that the larger the VFA, the higher the incidence of postoperative complications.

Abdominal VFA has been associated with a high incidence of multiple metabolic risk factors (21, 22) and it can be used as an indicator of visceral fat load in the postoperative review of weight-loss patients (23). Computerized abdominal tomography for assessing VFA has been widely used to predict these metabolic risk-related factors (24, 25). Abdominal CT in bariatric surgery is typically used for detecting postoperative complications (26). Although there is no consensus regarding routine CT-scanning before bariatric surgery, it can be used to guide the surgeon to determine the type of bariatric surgery to be offered to the patient and to evaluate the volumetric assessment of stomach and gastric sleeves in patients before and after bariatric surgery (27, 28). The concern associated with the use of CT is its radiation dose; however, it has been shown that, using the proper protocol, the average individual effective dose in bariatric patients is 7.8 mSv, which is not considerably higher than that used in an upper gastrointestinal study (29).

In this study, the total incidence of postoperative complications in laparoscopic bariatric surgery was 7.89%, and the incidence of serious complications (Clavien–Dindo grade ≥ III) was 3.29%. Complication rates reported in the present study were within the median range of complication rates in a previous study. In the previous global benchmark study, over 30 days, surgical outcomes showed that 5.5% (0.3 - 16.8) of SG patients presented any complication and 2% (0 - 9.7) presented complication grade ≥ IIIa after bariatric surgery (30). The overall complication rate in this study almost approached that in the previous global benchmark study (6.2% for sleeve gastrectomy). Regarding the incidence of postoperative complications, Ibrahim et al. (31) found that the incidence of serious postoperative complications widely varied among accredited bariatric surgery centers in the United States.

Increased VFA was associated with the occurrence of postoperative complications, which is consistent with the results of previous research on various disease types in multiple patient populations. In a study on 139 patients undergoing gastric cancer surgery and 110 patients undergoing colorectal cancer surgery, increased VAT area was associated with increased postoperative complications (32, 33). Another study on 2,100 patients evaluating body composition in relation to length of stay and postoperative outcomes found that increased VAT area was associated with an increased risk of readmission after colorectal cancer surgery (34). Visceral fat has a negative impact on prognosis, possibly because visceral fat can lead to a high incidence of hypertension, diabetes, and metabolic syndrome, thereby increasing cardiometabolic risk (21, 35). Evidence suggests an association between visceral fat and adverse effects across different disease types and surgical sites, supporting the notion that increased VFA is a marker of poor overall health. Obesity is excessive body fat, particularly visceral fat, which leads to changes in hormone levels, inflammation, and endothelial function (36). Patients with obesity are at a high risk of perioperative complications. Most importantly, obesity can cause difficulty establishing an artificial airway during anesthesia induction (difficult intubation or mask ventilation), atelectasis, or obstructive respiratory distress after extubation and is sometimes associated with residual opioids. OSA syndrome is significantly associated with obesity and increases the risk of perioperative and postoperative complications (37). Adipose tissue, particularly VAT, is an important metabolic tissue in the human body and has the characteristics of secreting harmful adipokines, leading to insulin resistance, chronic low-grade inflammatory response, and procoagulant states (38). Leptin is an adipokine produced by adipose tissue; physiological serum leptin levels are positively correlated with fat content. Obesity increases the amount of adipose tissue and serum leptin levels, thereby decreasing leptin sensitivity. It has been suggested that OSA pathogenesis is linked to leptin resistance. Animal studies have revealed that leptin-deficient mice have impaired respiratory function, reduced responsiveness to carbon dioxide, and hypoventilation when awake. Mice supplemented with leptin had increased sensitivity to carbon dioxide and increased ventilation per minute (39). Research indicates that visceral fat accumulation should be considered an important risk factor for OSA (40).

Owing to the accumulation of adipose tissue, small operating space under the endoscope, and vague surgical perspective of patients with obesity, it is difficult for surgeons to identify sufficient surgical planes and normal vascular systems (41). The pathophysiological mechanism between VAT and postoperative complication rate also remains unclear. First, high visceral fat may predispose patients to metabolic syndrome and cardiovascular disease; however, multivariate analysis limited the influence of these confounders as much as possible. Second, during the occurrence and development of obesity, white adipose tissue expands through hypertrophy and hyperplasia of white adipose cells (42). Adipokines such as leptin and adiponectin secreted by adipose tissue regulate insulin resistance, appetite, and obesity. Visceral obesity is related to leptin resistance, decreases adiponectin levels, decreases insulin sensitivity, and decreases insulin receptor number and affinity, thereby leading to the abnormal secretion of blood glucose, blood lipids, and hormones (43–45). VAT is sensitive to the stimulation of lipolysis, and fat cells release large amounts of free fatty acids, which aggravate insulin resistance (46). Perioperative metabolic disorders include insulin resistance, low-grade chronic inflammation, and abnormal insulin-like growth factor axis signaling, which can affect wound healing and patient prognosis and recovery.

Additionally, we observed a significant correlation between sex and postoperative complications in the univariate analysis; however, after age, sex, hypertension, and visceral fat factors were included in the multivariate analysis, sex, age and hypertension had no significance, and only VFA showed significance (p < 0.05). Therefore, VFA may be an independent risk factor for laparoscopic weight loss. The sex difference in fat distribution may be due to the regulatory effect of steroids.

Studies have shown that introducing a fast-track surgery program improves short-term outcomes (47). In this study, we did not fully adopt all ERAS programs, for example, in patients with obesity and diabetes, some ERAS measures were not implemented. Combined with unchanged medication, low-calorie intake may induce hypoglycaemia, while carbohydrate loading is associated with exaggerated hyperglycaemia. The construction of the ERAS program for patients with obesity, especially for those with underlying diseases, should differ from that of ordinary patients. The standardized implementation of ERAS should be prioritized for obese patients. Additionally, our center is gradually developing and implementing standardized protocols to improve the quality of care and reduce postoperative complications in accordance with the latest guidelines for perioperative care in bariatric surgery (48).

This study has some limitations. First, it was a single-center study conducted at our institution, with a BMI cut-off value of 28 kg/m^2^ recommended by the World Health Organization for obesity in Asian populations (49). Second, we only had partial follow-up data for a short time; therefore, long-term outcomes, such as survival, could not be determined. Long-term follow-up is required for future studies. Third, the study was a retrospective cohort study, which cannot control all confounding factors. It was difficult to obtain a large amount of clear and easy-to-measure preoperative imaging data. The sample size was also small, which may have caused selection bias. Fourth, because there are many provinces and cities in China, the population is large, and given the special characteristics of medicine in the country, retrospective research is difficult. Owing to its geographical location, our institution is an important academic center in Southwest China, and many patients travel from other cities for treatment. Patients from other provinces and cities are usually followed in local hospitals rather than our institution. This is the primary reason why some data were unavailable. Multicenter studies with larger sample sizes and longer follow-up periods are needed in the future. Given that our results showed an association rather than causation, further studies are needed to confirm the causal relationship between VFA and early postoperative complications.

## 5 Conclusion

VFA is an independent risk factor for early postoperative complications following bariatric surgery, and VFA may play a role in preoperative evaluation.

## Data availability statement

The original contributions presented in the study are included in the article/supplementary material. Further inquiries can be directed to the corresponding authors.

## Ethics statement

The studies involving human participants were reviewed and approved by Biomedical Ethics Review Committee, West China Hospital, Sichuan University. Written informed consent for participation was not required for this study in accordance with the national legislation and the institutional requirements.

## Author contributions

Conception and design: LH, CD, and XW. Provision of study materials: LH, CD, and XZ. Collection and assembly of data: LH, QW and CD. Data analysis and interpretation: LH, CD, and RZ. Manuscript writing: LH and CD. Manuscript revision: XW and YC. All authors contributed to the article and approved the submitted version.
